# The Structural Imbalance and Trajectory of Chinese National Policies on Medical–Preventive Integration: A Three-Dimensional Analysis of Policy Instruments (2015–2025)

**DOI:** 10.3390/healthcare14101372

**Published:** 2026-05-17

**Authors:** Wenjie Xu, Chi Zhang, Yuqi Yang, Xinyi Du, Yongze Zhang, Fang Wu

**Affiliations:** 1 School of International Pharmaceutical Business, China Pharmaceutical University, 639 Longmian Avenue, Nanjing 211198, China; 18341472816@163.com (W.X.);; 2 Admission and Employment Office, China Pharmaceutical University, 639 Longmian Avenue, Nanjing 211198, China

**Keywords:** medical-preventive integration, policy instrument, structural imbalance, LDA topic modeling

## Abstract

**Highlights:**

**What are the main findings?**

- Revealed a structural imbalance in policy instruments, where supply-side and environmental-side tools dominate, while demand-side mechanisms like health financing remain underutilized at only 14.61%.
- Identified a systemic neglect of rehabilitation and healthcare services, which together account for less than 30% of policy focus compared to the predominant “treatment-centric” orientation.

**What are the implications of the main findings?**

- Calls for a strategic shift toward demand-side instrument innovation, such as incentivizing private-sector participation and medical insurance reforms, to bridge the gap between service supply and public demand.
- Provides a decision-making framework for global health governance by demonstrating how smart health ecosystems and networked interagency collaboration can overcome institutional inertia in medical–preventive integration.

**Abstract:**

Background/Objectives: The global health landscape is currently confronted with dual challenges from both infectious diseases and chronic conditions. Medical–preventive integration has emerged as a pivotal strategy to address these issues, aiming to create a comprehensive, closed-loop framework that spans disease prevention, treatment, rehabilitation, and healthcare, ultimately improving population health outcomes. In the Chinese context, existing policies remain fragmented, scattered across various healthcare-related regulations, and lack systematic and comprehensive analysis. This policy fragmentation may impede the creation of synergistic effects essential for the effective implementation of integrated healthcare strategies. Methods: This study adopted a mixed-methods approach to analyze 85 national policies: a three-stage coding process identified 1088 policy nodes, and a three-dimensional framework (policy instruments (X) × full-cycle health service (Y) × integration stages (Z)) was applied to uncover systemic imbalances. Social network analysis and Latent Dirichlet Allocation (LDA) topic modeling were utilized to map interagency collaboration patterns and thematic shifts, which were visualized using Gephi and Sankey. Results: The analysis revealed that policy instruments are predominantly supply-side (45.04%) and environmental-side (40.35%), with demand-side instruments (14.61%) being notably underutilized, particularly in health financing. Rehabilitation services, representing just 8.27% of the policy focus, were identified as a significant gap in the comprehensive health service cycle. While 44.58% of the instruments facilitated collaboration of medical and preventive services, integration of medical–preventive management stagnated at 25.28%, reflecting institutional inertia that impedes the redistribution of cross-sector resources. Agency collaboration evolved from a siloed approach (2015–2018) to a networked structure (2019–2021) and transitioned to centralized governance post-2022. Thematic shifts in policy discourse moved from a “Healthy China” focus toward pandemic-driven disease surveillance, culminating in the recent development of smart health ecosystems. Conclusions: China’s policies for medical–preventive integration demonstrate notable structural imbalances, particularly in the economic instruments related to health financing and the private-sector participation in healthcare. These imbalances may impede the effective allocation of healthcare resources and hinder the seamless transition toward integrated care. Future policy efforts should focus on optimizing the structure of policy instruments, addressing gaps in the full lifecycle of health services, advancing integration reforms, and promoting the transformation of the healthcare system through enhanced collaborative governance among key stakeholders.

## 1. Introduction

Amid the accelerating global trend of population aging, imbalanced healthcare resource allocation, and the growing complexity of disease risks, medical–preventive integration has become a strategic priority in public health reforms worldwide [1,2]. Building upon established definitions of healthcare [3,4,5] and public health systems [6,7,8], the US Institute of Medicine (IOM) conceptualized medical–preventive integration as “linking primary care and public health through programs and activities to improve efficiency and population health outcomes” [9]. In 2018, the World Health Organization (WHO) classified integration models into six categories, based on workforce, service delivery, institutional alignment, training, and incentives [10]. Developed countries have pioneered innovative models, such as the US Program of All-Inclusive Care for the Elderly (PACE) [11] and UK Integrated Care Systems (ICSs), within which Integrated Care Boards (ICBs) act as statutory commissioning bodies [12]. In China, the Healthy China Initiative has shifted health priorities from disease treatment to a comprehensive approach that includes prevention, treatment, rehabilitation, and healthcare throughout an individual’s life. Within the Chinese context, “medical–preventive integration” refers to a system-wide provider integration. It extends beyond primary care to include public health departments in secondary/tertiary hospitals, as well as macro-level collaboration with Disease Control and Prevention. Thus, the policies analyzed in this study target the entire healthcare network to eliminate institutional silos. The 2015 National Medical and Health Service System Development Plan (2015–2020) first institutionalized integrated care, aiming to align medical and public health systems through governance restructuring, service integration, and incentive realignment [13]. While notable progress has been made in chronic disease management and the integration of traditional Chinese medicine [14], challenges persist in areas such as interagency coordination, workforce development, and sustainable financing. Fragmented policy frameworks remain a significant barrier, characterized by insufficient specialized regulations and a lack of comprehensive implementation guidelines [15,16].

Existing research on medical–preventive integration can be categorized into three primary domains. Firstly, studies on the effectiveness of policy implementation demonstrate, through randomized controlled trials (RCTs), that integrated services significantly improve quality of life for patients with cancer and chronic disease [17,18]. These services also contribute to advancing health equity [19,20] and transforming healthcare delivery systems [21,22,23]. Initially focused on chronic and infectious disease control, the scope of integrated services has since expanded to encompass whole-lifecycle health management [24]. Secondly, research on the factors influencing policy development highlights the role of power dynamics among health workers [25,26,27] and barriers to interdepartmental coordination [28,29] as major institutional obstacles. These challenges necessitate systematic planning in areas such as personnel mobility, information integration, and the establishment of incentive mechanisms [30,31]. Studies focusing on specific disease categories, such as HIV/AIDS and tuberculosis, emphasize the substantial impact of government investment on the integration of medical and preventive services [32,33]. Thirdly, in research on policy formulation itself, scholars predominantly utilize analytical frameworks based on dimensions such as policy instruments, service providers, and collaborative mechanisms. These studies identify structural imbalances and unclear hierarchical coordination in current policies, suggesting that strengthening top-level design and fostering multi-stakeholder collaboration could address these issues [34,35]. However, the scope of policy texts and analytical frameworks remains limited, with many studies focusing on institutional perspectives from healthcare providers rather than addressing broader population health needs. Methodologically, some scholars apply the PMC index model for quantitative evaluations of integrated healthcare–public health systems policies, but often without integrating multiple research methodologies. Furthermore, the predominant reliance on unidimensional, static perspectives has resulted in two key mismatches: (1) temporal discordance between policy formulation and the health service cycle phases, and (2) inadequate dynamic alignment between policy instrument selection and integration stages. Viewed through the lens of the Multiple Streams Framework (MSF), this temporal discordance essentially stems from the asynchronous convergence of the problem, policy, and political streams. Specifically, when emerging health service needs (the problem stream) lack concurrently mature policy solutions (the policy stream) or immediate institutional attention (the political stream), policy formulation inevitably lags behind practical demands. These theoretical and conceptual limitations hinder the ability of existing studies to capture the evolving nature of policy progression and to elucidate the complex interactions among multidimensional governance factors.

The purpose of this study is to conduct a comprehensive analysis of China’s policy on medical–preventive integration. This includes clarifying the proportional use and characteristics of policy instruments, systematically tracing the evolutionary logic of policy themes, addressing the persistent challenge of “integration without synergy” between medical and preventive services, and proposing targeted governance strategies to strengthen systemic policy development and fill existing gaps in policy perspectives. The goal is to provide decision-making insights for the transformation of global public health governance systems. To achieve this, the researchers constructed a three-dimensional analytical framework, “policy instruments–full-cycle health service–integration stages”, based on an in-depth investigation and analysis of relevant policies. This framework was employed to analyze the structural characteristics of policies and identify current priorities and directions. Apply LDA topic modeling to mine policy text data and explore thematic evolution trends. The study aims to address two key questions:

1. What are the release timeline, issuing authorities, and trends of China’s medical–preventive integration policies? What is the proportional use of policy instruments, and what internal logic underpins their application?
2. From the dual perspectives of the health service lifecycle and medical–preventive integration development stages, what are the distinctive features of policy instrument utilization, and what implications do these features have for policy formulation and optimization?

By addressing these questions, this research examines whether the structure and priorities of China’s medical–preventive integration policies are balanced and explores the logical starting points and evolutionary directions of these policies. The findings aim to enhance the scientific basis of policy design and contribute to the broader field of global health governance practices.

## 2. Materials and Methods

### 2.1. Information Sources

We systematically retrieved national-level policy documents (2015–2025) issued by the Central Committee of the Communist Party of China, the State Council, and the National Health Commission from three authoritative databases: the PKU Law Database, the Central People’s Government Portal, and the National Health Commission website. The search yielded 34, 30, and 21 preliminary documents from each source, respectively. To ensure the rigor of the policy corpus, a two-phase search strategy was employed: (1) Primary search: We targeted the core conceptual term “Medical–preventive integration” (Yi Fang Rong He, which refers to the structural and functional synergy between clinical medicine and public health services, aiming to break the institutional silos, identifying 181 foundational policies; and (2) expanded search: Given that Chinese administrative terminology evolved over the decade, a second phase was conducted using functional equivalents: “Medical-prevention coordination” (Yi Fang Xie Tong, focuses on the collaborative mechanisms and information sharing between clinical hospitals and Centers for Disease Control and Prevention), “Prevention-treatment integration” (Fang Zhi Jie He, a long-standing principle in Chinese health policy emphasizing that disease prevention should be integrated into the entire clinical treatment process), and “Public health” (Gong Gong Wei Sheng, included as a broader keyword to capture foundational systemic reforms that provide the institutional environment for integration). This expanded the collection to 425 documents. This dual-phase approach was essential to capture policies that address the same governance goals but utilize varying administrative phrasings across different periods or departments.

**Figure 1:**
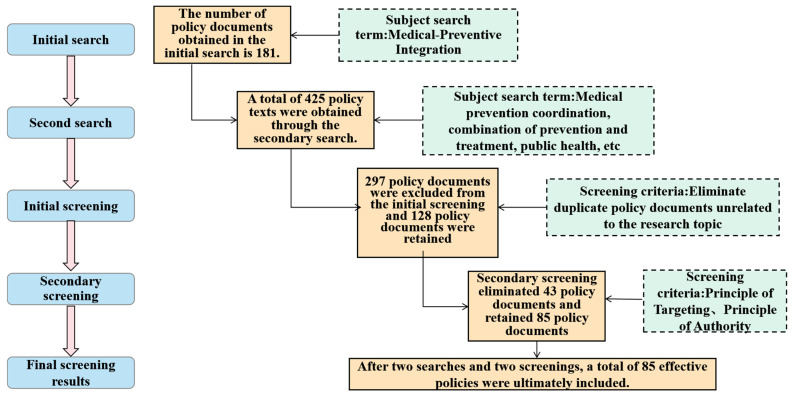
Document retrieval and screening process for integrated healthcare–public health policies.

Following a two-stage screening process (Figure 1), 85 policies met the inclusion criteria based on the following considerations: Relevancy: We excluded 297 documents that were unrelated to healthcare–public health integration; and authoritativeness: Only legally binding texts (guidelines, outlines, notices, and implementation opinions) were retained, while non-binding materials (speeches, official letters, and directives) and local regulations were excluded (Table 1). The inclusion criteria for policy documents were strictly defined based on administrative authority and functional relevance. In the Chinese governance system, although “Outlines”, “Guiding Opinions”, and “Notices” possess varying legal hierarchies, they collectively function as authoritative normative documents that dictate the strategic direction and operational protocols of health reforms. We included these diverse types to capture both high-level institutional blueprints and the granular implementation mandates necessary for systemic integration. The temporal boundary of 2015–2025 was selected because 2015 marked a critical institutional pivot—the issuance of the “National Healthcare Service System Development Plan Outline (2015–2020)”, which first systematically prioritized the integration of medical and preventive services. The timeline extends to 2025 to encompass the most recent “14th Five-Year” strategic cycle, providing a comprehensive longitudinal view of the policy trajectory. Please see Supplementary Material Table S1 for the detailed policy list.

### 2.2. Coding and Statistics

This study employed content analysis and manual coding using NVivo 12 software to systematically identify and categorize 85 policy documents. The analytical workflow proceeded as follows: Firstly, a three-dimensional analytical framework: A hierarchical coding system was established based on the “policy instruments × full-cycle health service × integration stages” framework. Subcodes were systematically assigned across the three dimensions and their constituent subcategories. Secondly, document coding protocol: Policy texts were coded following the sequential logic of “policy serial number–chapter identifier–sequence number–subcode designation”. This process resulted in the generation of 1088 discrete codes (Table 2). Thirdly, to ensure the reliability and objectivity of the manual classification of the 1088 policy nodes, two researchers (W.X. and YQ.Y.) independently coded the identified policy documents using NVivo 12. The inter-rater reliability was assessed using the weighted average Cohen’s kappa coefficient, which yielded a value of 0.843, indicating a high level of consistency between the coders. Any disagreements encountered during the coding process were resolved through in-depth discussions. If a consensus could not be reached, a third senior researcher (C.Z.) acted as an arbitrator to make the final determination, ensuring the rigor and consistency of the coding results.

To handle potential coding overlaps, complex policy sentences were disaggregated into atomic, mutually exclusive meaning units during the initial NVivo coding phase. Consequently, each of the 1088 identified meaning units was mapped to exactly one sub-category in the X-dimension, one in the Y-dimension, and one in the Z-dimension, thereby generating a unique (X, Y, Z) coordinate for each segment. This mutually exclusive coding protocol within every single dimension successfully prevented double-counting and ensured the statistical validity of the quantitative distribution.

### 2.3. Research Framework

To establish a clear analytic line from our research questions to final inferences, this study adopted a progressive analytical logic: First, a three-dimensional framework was applied to dissect ‘how’ policy instruments were structurally allocated to operationalize integrated care goals; second, social network analysis (SNA) was employed to map ‘who’ (the interagency network) governed and drove these policy agendas across different evolutionary stages; and finally, LDA topic modeling was utilized to uncover ‘what’ underlying thematic priorities and institutional logics emerged from this evolving policy discourse. This logical sequence ensures that our subsequent inferences regarding policy design and governance are tightly grounded in the multidimensional structure, collaborative patterns, and thematic content of the textual data.

**Figure 2:**
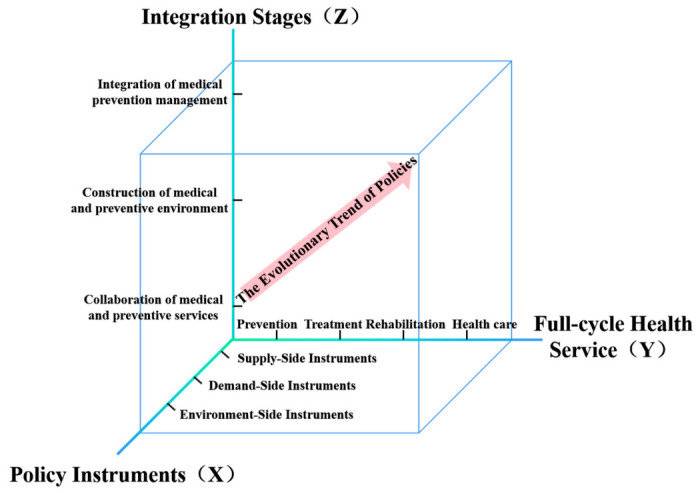
Three-dimensional analytical framework for integrated healthcare–public health policies.

From a policy logic perspective, the integration of health considerations into public policy was formally institutionalized as a core governance philosophy with the advancement of the Healthy China initiatives, underscoring the need for focused analysis on policy instruments and their operational efficacy [36]. Firstly, recognizing that governments rely on policy tools to drive the integration of healthcare and public health and to promote health equity, we adopt an instrument-centric approach to assess the effectiveness of policy implementation. Secondly, while China’s policy focus has shifted from a treatment-centric paradigm to one emphasizing full-lifecycle health services, persistent institutional inertia remains a challenge. As a result, there is a need for quantitative monitoring of policy prioritization, which is achieved through service cycle mapping. In parallel, our study integrates dynamic policy phase analysis, culminating in the development of a three-dimensional analytical framework (“policy instruments × full-cycle health service × integration stages”) (Figure 2). This framework allows for a comprehensive understanding of policy evolution and its alignment with the full health service lifecycle and integration stages.

**Figure 3:**
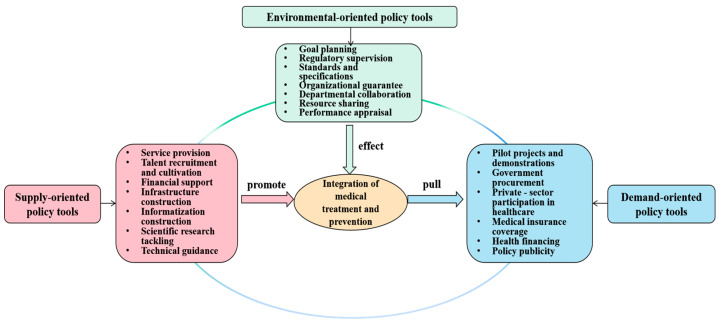
Taxonomy framework of policy instruments.

First, we discuss the X-dimension concerning the policy instruments. This study draws upon Rothwell and Zegveld’s [37] tripartite classification of policy tools, categorizing them into three types: supply-side, environmental-side, and demand-side policy instruments (Figure 3), comprising: Supply-side instruments: These are government-provided resources (such as funding, workforce, and infrastructure) aimed at directly advancing integration. They are operationalized through seven subcategories: service provision, talent recruitment and cultivation, informatization construction, financial support, infrastructure construction, scientific research tackling, and technical guidance. Demand-side instruments: These market-shaping mechanisms, which include insurance payment reforms, pilot programs, and public–private partnerships, are designed to stimulate societal demand. They are categorized into six sub-instruments: pilot projects and demonstrations, government procurement, private-sector participation in healthcare, medical insurance coverage, health financing, and policy publicity. Environmental-side instruments: These indirect enablers operate through regulatory frameworks and institutional environments. The subdomains include goal planning, regulatory supervision, standards and specifications, organizational guarantee, departmental collaboration, resource sharing, and performance appraisal (Table 3).

Next, we examine the Y-dimension concerning full-cycle health services. The Healthy China 2030 Planning Outline emphasizes two key focal points: serving all populations and covering the entire lifecycle. Its goal is to provide equitable, accessible, and systematic health services, including prevention, treatment, rehabilitation, and health promotion. Based on this policy framework, this study categorizes full-cycle health services into four distinct phases: Prevention: Proactive measures taken before disease onset to reduce risks, such as disease screening and risk prediction, aiming to minimize disease-inducing factors. Treatment: Medical interventions administered after disease onset to alleviate symptoms, cure diseases, or control progression. This phase encompasses clinical examinations, pharmacotherapy, and surgical procedures. Rehabilitation: Post-treatment efforts to restore patients’ physical functions, psychological well-being, and social adaptability. This phase includes postoperative training, elderly care, and psychological rehabilitation. Healthcare: Strategies aimed at consolidating health outcomes, enhancing resilience, and sustaining physical and mental wellness. This includes regular health checkups, traditional Chinese medicine (TCM) practices, and health management.

Finally, we analyze the Z-dimension, focusing on the developmental stages of medical–preventive integration. This study classifies the integration process into three progressive phases: Collaboration of medical and preventive services. This phase involves initial collaborative projects between disease control centers and medical institutions, such as the integration of traditional Chinese and Western medicine and research innovation. However, the integration at this stage is limited, with insufficient coordination in personnel, information sharing, and workflows. Construction of medical and preventive environment: This phase focuses on building external conditions that are conducive to integration. Key components include talent exchange programs, the development of digital health platforms, and the creation of a supportive sociocultural environment for collaboration. Integration of medical–preventive management: This phase involves the systematic alignment of institutional mechanisms, operational protocols, and resource allocation between disease control and medical services. Notable examples include the establishment of performance metrics for preventive services within medical institutions and the integration of preventive responsibilities into healthcare evaluation systems.

## 3. Results

### 3.1. Policy Structural Characteristics

#### 3.1.1. Single-Dimensional Analysis: Policy Instruments

**Figure 4:**
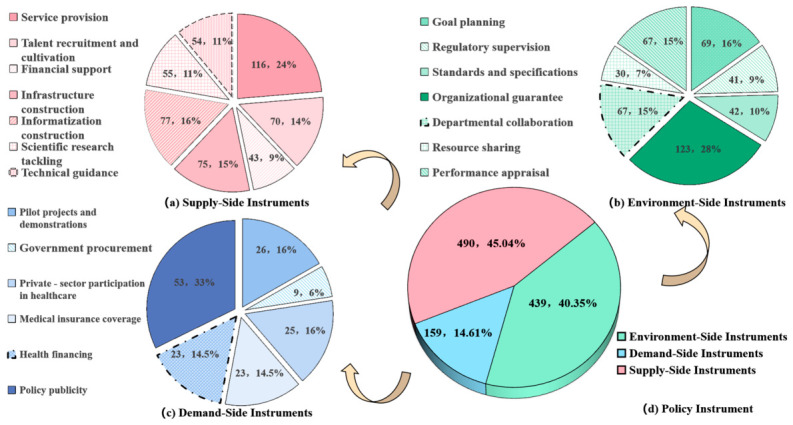
Distribution of overall utilization of policy instruments.

The study analyzed 1088 coding units from 85 policy texts (Figure 4). Supply-side instruments dominated the policy landscape, accounting for 490 instances (45.04%), followed closely by environmental-side instruments at 439 instances (40.35%). In contrast, demand-side instruments were underutilized, with only 159 instances (14.61%).

Further analysis revealed distinct patterns within each category: among supply-side instruments (Figure 4a), service provision emerged as the most frequently employed measure, with 116 applications (24%), while financial support was the least utilized, with only 43 instances (9%). Other supply-side instruments, including technical guidance, scientific research tackling, informatization construction, and infrastructure construction, showed comparable utilization levels. In contrast, demand-side instruments (Figure 4b) exhibited a heavy reliance on policy publicity, which accounted for 53 instances (33%), while government procurement was the least utilized, with only nine instances (6%). Other demand-side instruments, including pilot projects and demonstrations, health financing, private-sector participation in healthcare, and medical insurance coverage, showed similar usage frequencies. The environmental-side instruments showed a strong emphasis on organizational guarantees, with 123 instances (28%), while regulatory supervision (42 codes, 10%), resource sharing (41 codes, 9%), and standards and specifications (30 codes, 7%) appeared comparatively underdeveloped (Figure 4c).

#### 3.1.2. Cross-Dimensional Analysis: Policy Instruments (X)–Full-Cycle Health Service (Y)

**Figure 5:**
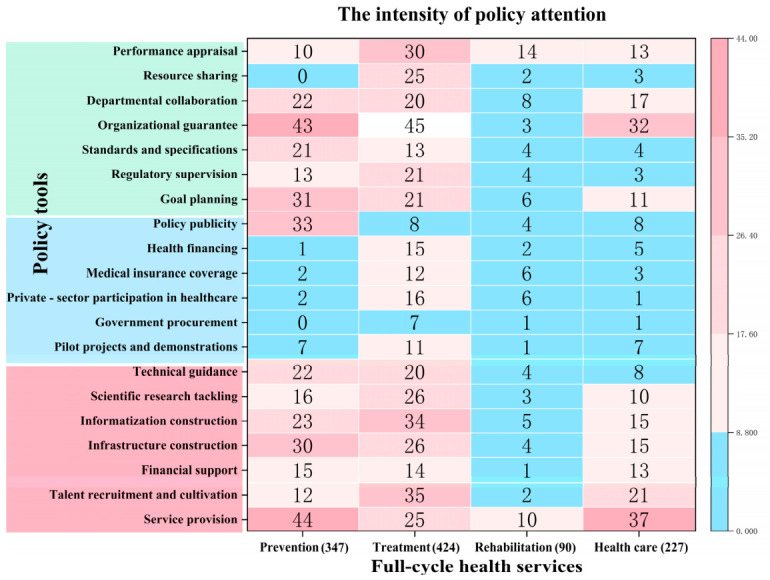
Bivariate distribution of policy instruments across full-cycle health service stages.

Through cross-dimensional analysis of the X-Y framework, the study highlights significant policy preferences across different health service domains (Figure 5). Treatment services dominate policy priorities, with policy instruments most intensively deployed in this area, totaling 424 instances (38.97%). This is followed by prevention services, which account for 347 instances (31.89%). Healthcare services receive comparatively less attention, with 227 instances (20.86%), while rehabilitation services exhibit the lowest level of policy engagement, with only 90 instances (8.27%). These findings suggest a persistent disease-treatment-centric policy orientation and point to a systemic neglect of rehabilitation services.

Further analysis of policy instrument imbalances is visualized through heatmaps, with blue indicating underutilization (frequency < 10) and red representing active application (frequency ≥ 10). First, prevention services predominantly rely on supply-side (46.69%) and environmental-side instruments (40.35%), with demand-side instruments (12.96%) remaining underutilized, particularly in market incentives. Furthermore, among supply-side instruments, infrastructure construction and informatization construction are prioritized over talent recruitment and cultivation, exacerbating the gaps in prevention services when compared to treatment-focused policies. Second, healthcare services exhibit a relatively balanced allocation of policy instruments, but there are internal structural disparities. Supply-side instruments emphasize service provision (37 codes) and talent recruitment and cultivation (21 codes), while financial support (13 codes) and technical guidance (10 codes) are insufficient. Environmental-side instruments primarily focus on organizational guarantees (32 codes) and departmental collaboration (17 codes), with limited adoption of resource sharing (three codes) and standards and specifications (four codes), thus constraining service coverage and efficacy. Third, rehabilitation services face significant deficits in both supply-side and environmental-side instruments. Only service provision and performance appraisal are moderately utilized, while all other tools register fewer than 10 applications, highlighting a systemic policy neglect in this domain.

#### 3.1.3. Cross-Dimensional Analysis: Policy Instruments (X)–Integration Stages (Z)

**Figure 6:**
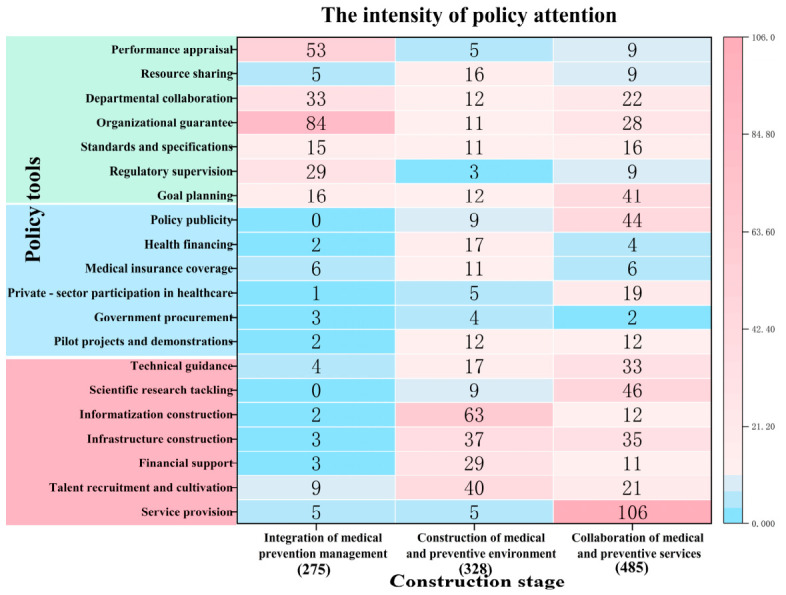
Bivariate distribution of policy instruments by integration stage.

Through cross-analysis of X-Z, the study reveals that the integration between medical institutions and public health agencies predominantly remains confined to operational coordination (Figure 6). Specifically, policy instruments were most frequently employed during the collaboration of medical and preventive services, with a total of 485 codes (44.58%), followed by the construction of a medical and preventive environment, which accounted for 328 codes (30.15%). The integration of medical–preventive management demonstrated the lowest utilization of policy instruments, with only 275 codes (25.28%). The lower frequency of policy instruments in the ‘Management Integration’ stage (25.28%) reflects both the inherent nature of this phase and a genuine policy gap. Inherently, management integration focuses on institutional consolidation and top-level design, which naturally requires fewer, broader policy instruments compared to active intervention stages that demand numerous specific guidelines and incentives. However, this low frequency also indicates a real gap: while macro-level structures have been established, there is a distinct lack of specific, operational policy tools designed to enforce and sustain actual collaboration between medical and public health institutions at the local level.

Further analysis of the underlying causes reveals three key patterns. Firstly, during the collaboration of medical and preventive services, supply-side instruments (54.43%)—such as service provision (106 instances) and technical guidance (33 instances)—predominate, reflecting a policy preference for advancing integration through resource allocation and technical support. However, this “technocratic governance” approach underscores institutional inertia, as evidenced by the underutilization of tools like performance appraisal and medical insurance coverage. Secondly, while demand-side instruments such as pilot projects, demonstrations, and health financing gained relatively more attention and appeared more frequently during the construction of the medical and preventive environment phase, their overall proportion and absolute policy support still remained insufficient compared with supply-side and environmental-side instruments. This was especially evident in the use of environmental instruments (17.68%), such as performance appraisal and standards and specifications, which failed to effectively connect operational coordination with administrative integration, ultimately limiting systemic effectiveness. Thirdly, in the integration of the medical–preventive management phase, there was an over-reliance on environmental instruments (85.45%)—such as organizational guarantees and departmental collaboration—while demand-side (5.09%) and supply-side (9.46%) instruments were largely neglected. This imbalance reflects the lag in institutional development relative to practical needs.

### 3.2. Evolutionary Trajectories of Integrated Healthcare–Public Health Policies

#### Policy Stage Delineation and Interagency Collaboration Network Dynamics

**Figure 7:**
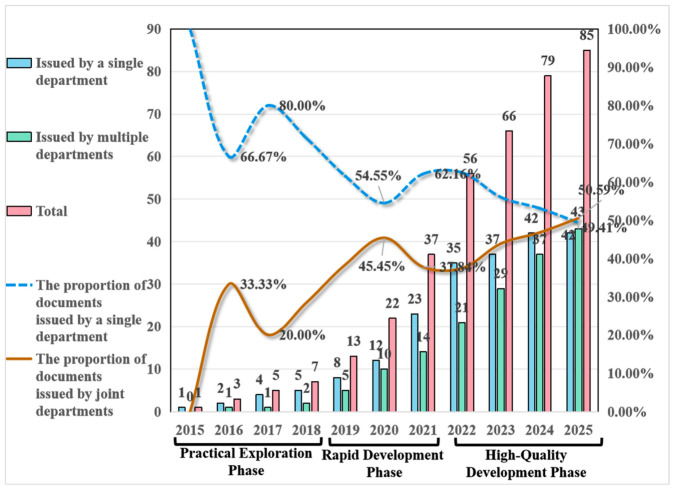
Temporal distribution of integrated healthcare–public health policy issuance.

Through quantitative, temporal, and institutional analyses of 85 policy documents related to medical–preventive integration, this study identifies three key trends in policy development since 2015: a consistent increase in attention, an accelerated frequency of policy issuance, and ongoing multi-departmental collaboration. Building on this statistical analysis, the research also employed co-occurrence analysis, combined with Gephi visualization, to explore the collaborative relationships among policy subjects (Figure 7). This approach aims to reveal the evolutionary patterns of inter-organizational coordination and assess their potential impacts, thus providing both theoretical foundations and practical insights for optimizing integrated healthcare–public health systems policies. In the visualization, node size corresponds to the frequency with which policy subjects appear, line thickness reflects the frequency of co-occurrences, and node centrality represents the intensity of inter-subject connections. A comprehensive analysis of policy statistics and the evolution of collaboration among issuing institutions reveals that the development of China’s integrated healthcare–public health systems policies can be divided into three distinct phases.

**Figure 8:**
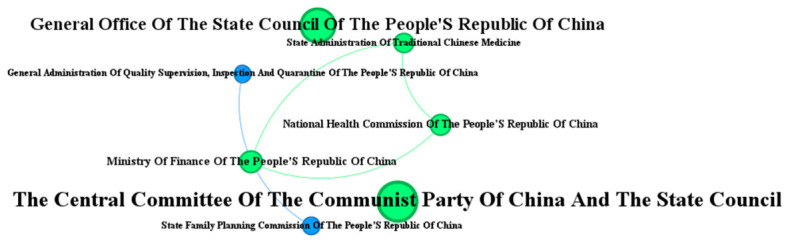
Network of issuing agencies in the practical exploration phase (2015–2018).

During the initial practical exploration phase (2015–2018), policy formulation exhibited a low frequency (averaging 3.5 documents annually), slow growth (with only two new documents per year), and limited departmental involvement (seven issuing departments). These early efforts were primarily based on standalone documents issued by the CPC Central Committee and the State Council, lacking effective interdepartmental coordination (Figure 8). Following the market-oriented reforms of 1979, China’s healthcare system experienced significant fragmentation between medical treatment and disease prevention. This fragmentation persisted until 2015, when the concept of “prevention–treatment integration” was first proposed. The 2016 “Healthy China 2030” Planning Outline introduced a visionary “trinity” mechanism, combining public health institutions, general and specialty hospitals, and primary healthcare facilities, thereby initiating the transformation of the service delivery model. By 2018, the Notice on Implementing Family Doctor Contract Services formally mandated “integrated Medical–Preventive services” (Yi Fang Rong He), marking the first official and explicit emergence of the integrated care concept in operational policy documents.

**Figure 9:**
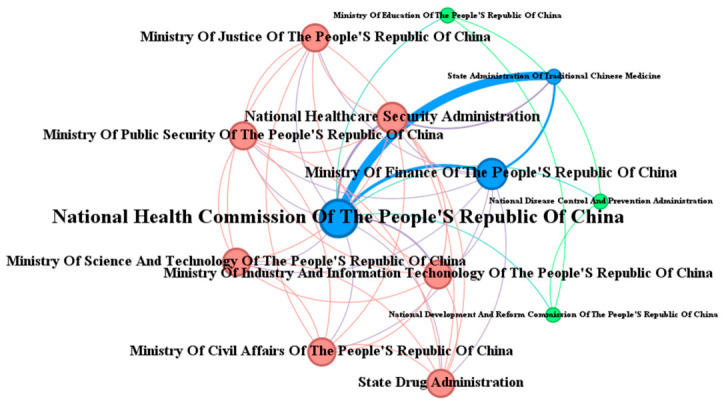
Network of issuing agencies in the rapid development phase (2019–2021).

The second phase, marked as the rapid development stage (2019–2021), was significantly influenced by the COVID-19 pandemic, which highlighted the critical importance of public health initiatives and accelerated the integration of medical and preventive services. This acceleration was evident in the increased frequency of policy issuance, with the annual average rising from 3.5 to 10 documents, accompanied by a higher growth rate of approximately 70% per year. Additionally, the 2019 Law of the People’s Republic of China on Basic Medical Care and Health Promotion, which mandated “integrating health concepts into all policies and prioritizing prevention”, facilitated enhanced interdepartmental coordination. Consequently, the primary issuing bodies shifted from predominantly standalone documents by the State Council to multi-department collaborations centered around the National Health Commission (NHC) and the National Healthcare Security Administration, evolving from a “single-line collaboration” model to a “networked collaboration” framework (as illustrated in Figure 9). However, persistent challenges remained during this phase, including delayed information dissemination, inefficient cross-departmental communication channels, and unclear jurisdictional boundaries. These issues continued to threaten the coherence and effectiveness of policy implementation. Addressing these challenges requires urgent mitigation through optimized institutional design, strengthened interdepartmental data sharing, and clearer responsibility demarcation. In response to these issues, subsequent policies, such as the 2022 14th Five-Year Plan for National Health, have explicitly emphasized the need for further structural improvements.

**Figure 10:**
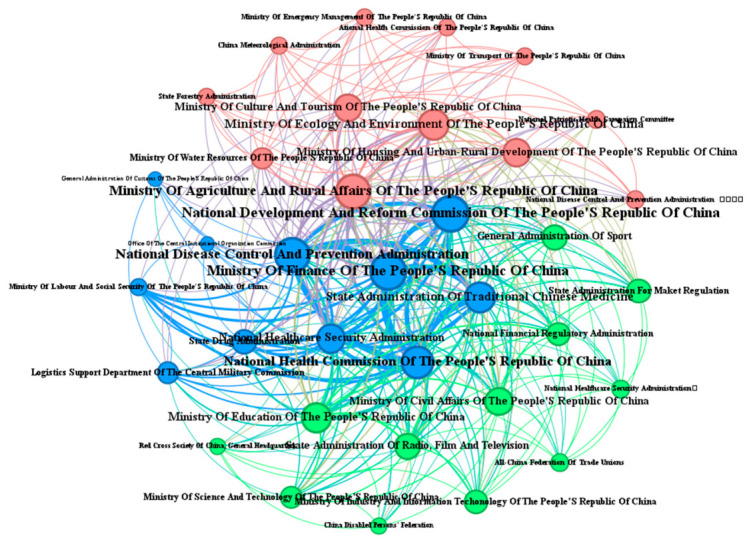
Network of issuing agencies in the high-quality development phase (2022–2025).

The third phase, referred to as the high-quality development phase (2022–2025), began in December 2022 with the normalization of COVID-19 prevention and control under “Class B infectious disease management”. During this phase, the frequency of document issuance increased to an average of 14 documents annually, while the growth rate slowed to approximately 20% per year (Figure 7). Research indicates a strategic shift in focus, prioritizing the quality of documents over their quantity. The number of participating institutions expanded significantly, reaching 35 entities, with a predominant adoption of multi-departmental joint issuance mechanisms. This led to the establishment of a three-tiered “intensive collaborative” framework, comprising six core departments, including the National Health Commission and the Ministry of Finance; eleven secondary centers, such as the Ministry of Education and the National Medical Products Administration; and eighteen peripheral participants, including the General Administration of Customs and the China Meteorological Administration (Figure 10). Notably, the marginalization of agencies like the General Administration of Customs could create governance gaps in cross-border infectious disease prevention, potentially conflicting with global health governance trends. A defining feature of this phase is the detailed planning and implementation of innovative medical–prevention integration mechanisms. Document formats transitioned from simple notifications to more comprehensive guidelines. For example, the Guidelines on Promoting High-Quality Development of Disease Prevention and Control outlined specific measures, including the establishment of compact county-level medical consortia, health village initiatives, talent development programs, and technological innovation requirements. These initiatives emphasize both institutional innovation and technical advancement, marking a significant shift towards a more structured and strategic approach to healthcare–public health integration.

To quantitatively validate the network structure and the trajectory of centralized governance, we calculated the degree and betweenness centrality for key nodes. Notably, the National Health Commission (NHC) demonstrated a significant expansion in its collaborative network over time. Its degree centrality consistently increased from 0.333 in the first stage to 0.545 in the second stage, and reached 0.750 in the third stage. This indicates that by the final stage, the NHC co-issued policies with 75% of all involved departments. Furthermore, its betweenness centrality peaked during the second stage (0.125), highlighting its crucial bridging role in linking disparate governmental bodies. These specific centrality metrics robustly corroborate the visual representation of an increasingly centralized and cohesive interdepartmental governance structure.

### 3.3. Temporal Shifts and Institutional Logics of Policy Themes

This study employs the Latent Dirichlet Allocation (LDA) model, which offers three distinct advantages in policy text analysis. Firstly, by constructing a three-dimensional semantic network of “vocabulary–topic–document” through its probabilistic generative model, the LDA model overcomes the path dependency on explicit keywords that is characteristic of traditional content analysis methods. This enables the model to capture implicit semantic association patterns within policy texts. Secondly, leveraging its unsupervised learning mechanism based on the Dirichlet prior distribution, the model autonomously identifies latent structures of policy themes without relying on predefined classification frameworks. This makes it particularly suitable for analyzing multidimensional and composite texts, such as China’s medical–prevention integration policies. Furthermore, the temporal dynamic comparability of topic probability distributions generated by the model allows for the systematic characterization of policy evolution features, providing valuable methodological support for longitudinal comparative studies of health policy development trajectories. For text preprocessing, the Jieba tool was utilized for Chinese text segmentation. The preprocessing pipeline included standard tokenization, removal of punctuation, and the elimination of noise using a customized Chinese stopword list. In configuring the Latent Dirichlet Allocation (LDA) models, our analysis accounted for the dynamic evolution of policies across three distinct stages. The optimal number of topics (K) for each stage was independently determined by comprehensively evaluating both perplexity and coherence scores across a tested range of K = 1 to 16. Consequently, the optimal topic numbers were identified as K = 5, K = 7, and K = 8 for the first, second, and third stages, respectively. To optimize model performance dynamically, the hyperparameters for document–topic density and topic–word density were both set to be calculated automatically by the algorithm, ensuring the optimal distribution specific to each stage’s corpus. Following the automated generation of the topic clusters, the specific semantic labels for each topic were assigned interpretively by the research team, based on the highest-probability terms within each corresponding cluster.

**Figure 11:**
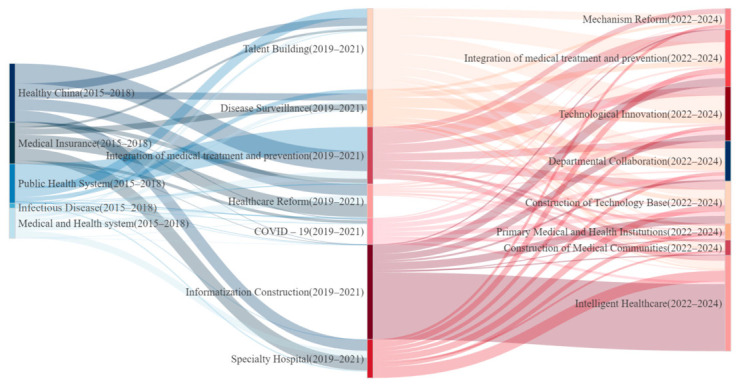
Sankey diagram of thematic evolution in integrated healthcare–public health policies.

The study reveals that the thematic evolution of medical–preventive integration policies underwent two significant transitions over the past decade. The first transition occurred between the practical exploration phase (2015–2018) and the rapid development phase (2019–2021), marked by a shift from “conceptual advocacy” to “practical exploration” (Figure 11). During the practical exploration phase, policy themes predominantly focused on “Healthy China” and “medical insurance”, with the medical and public health systems remaining separate. This reflected the embryonic nature of medical–preventive integration concepts at this stage. By the rapid development phase, the “Healthy China” theme had undergone structural fission, evolving into practical implementation themes such as talent development, specialty hospitals, and healthcare reforms. Ultimately, this marked the merging of medical and public health systems into a new integrative theme. The second transition occurred between the rapid development phase (2019–2021) and the high-quality development phase (2022–2025), characterized by an evolution from “infrastructure construction” to “smart system development.” While the COVID-19 theme gradually diminished during the high-quality development phase, the transition from emergency governance to normalized governance was facilitated through smart system optimization, rather than simple regression. This institutional upgrade manifested through technological advancements, with the theme of “informatization construction” further evolving into “intelligent healthcare.” This shift not only represents a technical enhancement but also signifies a conceptual elevation of policy objectives. It emphasizes intelligent integration through advanced information technologies, aiming to establish unified medical–preventive service platforms and management systems that optimize resource allocation, refine service delivery, and enable intelligent disease surveillance. Collectively, these measures aim to comprehensively enhance the quality and efficiency of medical–preventive integration, better meeting the evolving demands of public health. Additionally, the differentiated themes of “departmental collaboration” and “construction of medical communities” illustrate policy refinement through concrete implementation strategies, further indicating the maturation of integrated healthcare–public health systems policies.

## 4. Discussion

This study constructs a three-dimensional analytical framework—comprising “policy instruments”, “health service cycles”, and “integration stages”—to quantitatively evaluate 85 policies related to medical–preventive integration from 2015 to 2025. By employing social network analysis of policy issuers and Latent Dirichlet Allocation (LDA) topic modeling, the study identifies thematic trends across various stages, thereby overcoming the limitations of single evaluation methods. The study findings revealed that China’s medical–preventive integration policy faces structural imbalances in policy instruments and superficial integration of healthcare services, which may ultimately lead to structural mismatches in health resource distribution. Systematic integration of policy frameworks is therefore imperative, encompassing both horizontal cross-sectoral collaboration—such as joint resource allocation between health, finance, and medical insurance departments—and vertical hierarchical alignment to ensure that macro-level blueprints are effectively translated into local implementation.

Regarding policy instruments, the overall structure reveals an imbalance, with a predominant reliance on supply-side (45.04%) and environmental-side instruments (40.35%), while demand-side instruments are insufficiently utilized (14.61%). While this overall quantitative imbalance aligns with the findings of prior two-dimensional analyses, such as those by Li Yilin and Xiong Zihui [38], our three-dimensional framework significantly advances the theoretical understanding of this phenomenon. Traditional two-dimensional models primarily capture a static snapshot of instrument distribution across health service targets. In contrast, by introducing ‘integration stages’ (the Z-dimension) as an evolutionary axis, our framework transcends static observation to map the dynamic trajectory of policy deployment. It reveals that the heavy reliance on supply-side and environmental-side instruments is not merely a quantitative artifact, but a structural feature deeply embedded in China’s phased approach to integrated care, where early stages necessitate direct resource provision and institutional environment-building, historically neglecting the stimulation of demand-side forces [39]. Consequently, there is a pressing need to reduce the reliance on supply and environmental tools while enhancing the use of demand-side instruments. Within supply-side instruments, service provision is dominant, as exemplified by the 2024 Notice on Basic Public Health Services [39], which promotes multi-disease prevention and management for chronic conditions. However, financial support remains minimal, lacking dedicated funding mechanisms, which hinders the potential impact on health outcomes [40]. On the environmental side, tools like regulatory supervision, resource sharing, and interdepartmental coordination are underutilized, reflecting fragmented governance structures [41]. In terms of demand-side instruments, there is a notable absence of government procurement, health financing innovations, and private capital engagement, leading to an underutilization of market forces [42]. To address these issues, the study recommends: reducing redundant service supply (e.g., repetitive family doctor contracting policies); increasing fiscal allocations that are integrated with demand-side instruments; establishing unified service standards and performance metrics; and incentivizing private-sector participation to better align supply and demand dynamics.

From the perspective of the health service cycle, policy instruments disproportionately focus on treatment rather than rehabilitation and healthcare. Prevention strategies tend to rely heavily on awareness campaigns, while neglecting critical areas such as talent recruitment and cultivation, and performance incentives. To address these gaps, it is recommended that governments increase funding for prevention, promote resource sharing, and establish integrated health information platforms [43]. Regarding integration stages, collaboration between medical and preventive services is prioritized over managerial integration, reflecting institutional inertia [44]. Proposed solutions to this issue include establishing dedicated leadership teams, standardizing performance evaluations, and implementing personnel exchange programs.

Policy evolution analysis reveals three distinct phases: exploratory (2015–2018), rapid development (2019–2021), and high-quality development (2022–2025). During these phases, agency collaboration evolved from siloed efforts to multi-sectoral coordination. The increased involvement of ministries aligns with the principles of “Health in All Policies” (WHO, 2021), but challenges such as inefficiencies and ambiguities in prioritization continue to persist. The thematic progression from the “Healthy China” initiative to disease surveillance and innovation highlights a shift from theoretical concepts to practical implementation.

The findings suggest that the fundamental bottleneck in China’s medical–preventive integration is not a lack of policy attention, but rather the structural gaps in governance design. While the high frequency of environmental and supply-side instruments reflects strong governmental commitment, the systemic “imbalance”—characterized by weak demand-side incentives and fragmented interagency collaboration—indicates that the formal governance framework has yet to translate policy intentions into functional synergy. Therefore, policy optimization should shift from merely increasing “policy volume” to refining governance quality, specifically by strengthening cross-sectoral leadership mechanisms and innovating health financing structures to bridge the institutional silos between clinical and preventive services. To address these challenges and enhance policy effectiveness, governments should: (1) conduct forward-looking planning for emerging technologies, (2) establish mechanisms for monitoring policy effectiveness, and (3) phase out outdated measures that no longer serve their intended purpose.

To fulfill the proposition of providing a decision-making framework for global health governance, it is essential to contextualize China’s policy instrument imbalances within broader international trends. China’s predominant reliance on supply-side and environmental-side instruments mirrors the transitional challenges frequently observed in other BRICS nations, such as India and Brazil. These developing health systems often grapple with institutional fragmentation and a historical dependence on direct government provision, which tends to marginalize market-based and patient-driven incentives. In contrast, many OECD countries have successfully leveraged demand-side instruments to catalyze medical–preventive integration. For instance, healthcare models in the United Kingdom and the United States increasingly utilize value-based purchasing, sophisticated social health insurance reforms, and targeted tax incentives to stimulate private-sector engagement and foster patient-centered integrated care. Consequently, China’s imperative to diversify its demand-side mechanisms—particularly through innovative health financing and strategic purchasing—offers a universal lesson for developing health systems worldwide: achieving genuine medical–preventive integration requires moving beyond mere structural resource provision (supply) to actively incentivizing collaborative behaviors and systemic efficiency (demand).

## 5. Conclusions

This study focuses on China’s unique reform model for optimizing health resource allocation—the medical–preventive integration system. Through the lens of policy instrument analysis, it delves into the structural challenges and evolutionary trends within its policy framework. The findings reveal that despite significant progress in building the integrated system, structural imbalances persist. Medical services have not yet achieved full coverage across the entire lifecycle, while the integration of medical and preventive care remains superficial, lacking managerial-level consolidation. These issues reflect structural conditions that may exacerbate potential health inequities, requiring continuous attention. To achieve enhanced collaborative governance, policymakers should increase the application of demand-side policy instruments and strengthen cross-sectoral management. Specifically, we recommend establishing a dedicated medical–preventive integration commission at both national and provincial levels—conceptually analogous to the Integrated Care Boards (ICBs) in the United Kingdom—to break down institutional silos, enforce joint accountability, and propel China’s healthcare system toward higher efficiency and functional integration.

It is important to acknowledge the role of the COVID-19 pandemic during the rapid development phase (2019–2021). While policies during this period exhibited characteristics of emergency governance, they should not be viewed merely as temporary anomalies. Instead, the pandemic acted as a fundamental institutional catalyst for standard integration efforts—accelerating the establishment of the National Disease Control and Prevention Administration and institutionalizing the long-term strategy of “peacetime and emergency integration”. Consequently, the instrument imbalances observed during this period reflect a permanent structural entrenchment rather than a transient crisis response.

While this study has revealed several critical issues in the field, it is essential to acknowledge its limitations. First, the research focused solely on national-level integrated medical and preventive care policies. Future studies could extend this work by systematically reviewing provincial policies to compile a comprehensive policy collection and investigate their implementation outcomes. Second, although the current analysis evaluated existing challenges through the lens of policy instruments, there is a pressing need to assess policy effectiveness from the patient perspective. Specifically, further investigation should explore how patients’ health literacy influences the impact of integrated medical and preventive care policies. Third, this study relies fundamentally on policy text analysis. It must be acknowledged that the frequency of coded policy segments and the volume of policy issuance reflect formal governance priorities, intended structural designs, and thematic evolution, rather than being direct measures of local implementation intensity or actual integrated service delivery outcomes. Consequently, our findings should be strictly interpreted as an analysis of national policy design and institutional logic, rather than a direct evaluation of policy effectiveness.

## Figures and Tables

**Table 1:** National integrated healthcare–public health policy documents (Selected), 2015–2025.

No.	Policy Title	Issuance Date
01	Notice on Issuing the National Healthcare Service System Development Plan (2015–2020)	30 March 2015
02	Healthy China 2030 Plan Outline	25 October 2016
03	Mid-term Evaluation Report on China’s National Malaria Elimination Action Plan (2010–2020)	18 August 2016
04	Notice on Issuing the Medium- to Long-Term Plan for the Prevention and Treatment of Chronic Diseases in China (2017–2025)	22 January 2017
05	Guiding Opinions on Promoting the Construction and Development of Medical Consortiums	26 April 2017
06	Notice on the Implementation of National Basic Public Health Service Programs in 2018	13 June 2018
07	Notice on the Provision of Family Doctor Contract Services in 2018	29 March 2018
08	Notice on Launching Pilot Projects for the Construction of Urban Medical Consortiums	16 May 2019
09	Basic Healthcare and Health Promotion Law of the People’s Republic of China	9 December 2019
…	…	…
82	Opinions on the Construction and Development of Interdisciplinary Strategic Talents in Medicine, Prevention, and Management	12 May 2025
83	Notice on Issuing the Action Plan for the Prevention and Treatment of Viral Hepatitis in China (2025–2030)	3 September 2025
84	Guiding Opinions on Strengthening Health Management Services for Chronic Diseases at the Primary Care Level	24 October 2025
85	Guiding Opinions on Strengthening the Construction of Specialized Departments in Primary Healthcare Institutions	11 December 2025

**Table 2:** Policy text coding matrix.

No.	Policy Title	Analyzed Text Segment	Code	Policy Instrument
1	Notice on Issuing the National Healthcare Service System Development Plan (2015–2020)	Chapter 6: Functional Integration and Division of Labor Collaboration Sec6.1-Prevention_Treatment_Integration. Professional public health institutions shall strengthen guidance, training, and assessment of public hospitals, primary healthcare institutions, and private hospitals in delivering public health services.	1-6-1-1	Supply-side instruments: Service provision
…	…	…	…	…
38	Notice of the General Office of the State Council on Issuing the Key Tasks for Deepening the Healthcare System Reform in 2022	Chapter 3: Strengthening Public Health Service Capacity. Strengthen medical–prevention collaboration. Advance implementation of high-risk screening and intervention programs for major chronic diseases (cancer, stroke, cardiovascular diseases, COPD). Promote pilot projects for integrated management of “triple-H” conditions (hypertension, hyperglycemia, hyperlipidemia), refine appropriate technologies and service models for chronic disease health management, and enhance integrated medical–prevention management of chronic diseases at the primary care level.	38-3-4-1	Demand-side instruments: Pilot projects and demonstrations
		Further implement the Healthy China Initiative. Solidly advance the Initiative by improving operational mechanisms to ensure achievement of phased targets by 2022. Continue to deepen the Patriotic Health Campaign. Establish performance appraisal mechanisms for health education and health promotion activities conducted by medical institutions and professionals. […]	38-3-4-2	Environmental-side instruments: Performance appraisal
…	…	…	…	…

**Table 3:** Policy instrument (X-dimension): Taxonomy and operational definitions.

Instrument Name	Instrument Type	Operational Definition in Integrated Healthcare–Public Health Systems
Supply-side instruments	Service provision	The government directly organizes or coordinates medical institutions and public health agencies to jointly provide integrated medical and preventive services.
	Talent recruitment and cultivation	The government attracts and cultivates interdisciplinary professionals with integrated medical and preventive capabilities through measures such as improving compensation, reforming academic institutions, and organizing training programs.
	Financial support	Direct fiscal inputs, subsidies, and capital injections provided by the government to support health programs or institutional operations.
	Infrastructure construction	Provision of physical facilities, tangible hardware, and spatial resources. This strictly refers to “brick-and-mortar” investments, such as the construction of hospital buildings, laboratory physical spaces, and the procurement of traditional medical equipment.
	Informatization construction	Development of digital platforms, software systems, and data-sharing networks. Any intervention related to digital infrastructure, such as Electronic Health Records (EHR), telemedicine networks, cloud computing, and big data analytics, is exclusively categorized here.
	Scientific research tackling	The government encourages institutions and universities to advance technological innovations in pharmaceuticals, medical practices, and digital solutions across the entire lifecycle of disease prevention, treatment, rehabilitation, and healthcare.
	Technical guidance	The government employs administrative measures to promote collaboration among medical institutions and public health agencies and enhance the capabilities of integrated health and prevention services.
Demand-side instruments	Pilot projects and demonstrations	The government employs a policy approach of establishing pilot cities to conduct preliminary trials in specific regions, exploring replicable implementation models for the integration of medical care and prevention, thereby guiding broader policy diffusion through targeted policy instruments.
	Government procurement	The government procures integrated medical and preventive services from social organizations or market institutions through fiscal funds.
	Private-sector participation in healthcare	The government implements policies such as market entry deregulation and tax incentives to encourage private capital investment in establishing diversified health service institutions, including medical facilities, rehabilitation centers, and integrated medical-care facilities. These entities serve as a complementary component to the public healthcare system, jointly participating in integrated medical and preventive services.
	Medical insurance coverage	Specifically refers to the demand-side reimbursement mechanisms, including the expansion of the insurance catalog, reimbursement ratios, and payment security for patients.
	Health financing	Refers to the broader resource mobilization framework, including multi-channel funding, private capital participation, and the systemic allocation of health expenditures.
	Policy publicity	The government popularizes and promotes the integrated healthcare and disease prevention policy by organizing conferences, public lectures, and related outreach activities.
Environmental instruments	Goal planning	The government conducts comprehensive planning and description of the integrated medical care and prevention-related work, and formulates specific implementation rules and detailed operational guidelines.
	Regulatory supervision	The government strengthens supervision of healthcare–prevention integration by enforcing mandatory measures including laws, regulations, departmental rules, and operational frameworks.
	Standards and specifications	The government establishes unified service standards, technical guidelines, and operational procedures to eliminate service heterogeneity, ensuring that integrated medical and preventive services provided by different institutions meet uniform quality requirements.
	Organizational guarantee	The government employs policy measures such as institutional setup, personnel allocation, and system construction to provide essential administrative support and resource allocation capabilities for the integrated medical and preventive services.
	Departmental collaboration	The government employs governance mechanisms such as joint meetings and collaborative document issuance to integrate resources and information across multiple departments, including health and wellness, medical insurance, finance, and education, thereby addressing institutional barriers and fragmentation of actions in the integration of medical and preventive services.
	Resource sharing	The government facilitates the interconnection and collaborative utilization of human resources, equipment, and data between medical institutions and public health agencies through institutional design and mechanism innovation.
	Performance appraisal	The government establishes a quantitative evaluation system to monitor process quality and health outcomes, with evaluation results directly linked to resource allocation.

## Data Availability

No new data were created or analyzed in this study.

## Supplementary Materials

The following supporting information can be downloaded at: https://www.mdpi.com/article/10.3390/healthcare14101372/s1, Table S1: Full List of the 85 National Policy Documents on Medical-Preventive Integration (2015–2025).
