# Supplementary material for: The Structural Imbalance and Trajectory of Chinese National Policies on Medical–Preventive Integration: A Three-Dimensional Analysis of Policy Instruments (2015–2025)
Source: Healthcare (Basel). 2026 May 17;14(10):1372. doi: 10.3390/healthcare14101372 (PMC13205401; doi:10.3390/healthcare14101372)
Supplement: Supplementary file 1 [file healthcare-14-01372-s001.zip › healthcare-4201381-supplementary.pdf]

Supplementary Table S1. Full List of the 85 National Policy Documents on Medical-Preventive Integration (2015–2025)

No.	Policy Title	Issuance Date
1	Notice on Issuing the National Healthcare Service System Development Plan (2015-2020)	2015.03.30
2	"Healthy China 2030" Plan Outline	2016.10.25
3	Mid-term Evaluation Report on China's National Malaria Elimination Action Plan (2010-2020)	2016.08.18
4	Notice on Issuing the Medium- to Long-Term Plan for the Prevention and Treatment of Chronic Diseases in China (2017-2025)	2017.01.22
5	Guiding Opinions on Promoting the Construction and Development of Medical Consortiums	2017.04.26
6	Notice on the Implementation of National Basic Public Health Service Programs in 2018	2018.06.13
7	Notice on the Provision of Family Doctor Contract Services in 2018	2018.03.29
8	Notice on Launching Pilot Projects for the Construction of Urban Medical Consortiums	2019.05.16
9	Basic Healthcare and Health Promotion Law of the People's Republic of China	2019.12.09
10	Notice on Professional Public Health Institutions such as County-Level Disease Control Centers Guiding Primary Institutions in Delivering Basic Public Health Services	2019.11.08
11	Notice on Further Promoting the Experience of Deepening the Healthcare System Reform in Fujian Province and Sanming City	2019.11.06
12	Notice on the Implementation of National Basic Public Health Service Programs in 2019	2019.08.30
13	Notice on the Provision of Family Doctor Contract Services in 2019	2019.04.23
14	Opinions on Strengthening the Construction of the National Health Information Standardization System	2020.09.27
15	Notice on Issuing the 2020 Work Plan for Promoting the Implementation of the Healthy China Initiative	2020.08.24
16	Notice on the Implementation of National Basic Public Health Service Programs in 2020	2020.06.12
17	Notice on Issuing the Administrative Measures for Medical Consortiums (Trial)	2020.07.09
18	Notice on Issuing the Key Tasks for Deepening the Healthcare System Reform in the Second Half of 2020	2020.07.16
19	Notice on Issuing the Evaluation Standards and Monitoring Indicator System for the Construction of Tight-Knit County Medical and Health Communities (Trial)	2020.08.31
20	Guiding Opinions on Accelerating the Innovative Development of Medical Education	2020.09.23
21	Recommendations of the Central Committee of the Communist Party of China on Formulating the 14th Five-Year Plan for National Economic and Social Development and the Long-Range Objectives Through 2035	2020.10.29
22	Notice on Deepening the "Internet + Healthcare" and "Five Ones" Service Action	2020.12.04
23	National Disability Prevention Action Plan (2021-2025)	2021.12.14
24	14th Five-Year Plan for Universal Medical Security	2021.09.23

25	Key Tasks for Deepening the Healthcare System Reform in 2021	2021.05.24
26	Opinions on Promoting the High-Quality Development of Public Hospitals	2021.05.14
27	Action Plan for Promoting High-Quality Development of Public Hospitals (2021-2025)	2021.09.14
28	Action Plan for Eliminating the Public Health Hazard of Hepatitis C (2021-2030)	2021.08.31
29	Guiding Plan for the Construction of Comprehensive Pilot Zones for Primary Health Care	2021.08.16
30	Notice on the Implementation of National Basic Public Health Service Programs in 2021	2021.07.12
31	Notice on Accelerating the Construction of Community Hospitals	2021.06.08
32	Notice on the Construction of High-Level Schools of Public Health	2021.12.16
33	Implementation Opinions on Deeply Promoting the Experience of Sanming City, Fujian Province, and Deepening the Healthcare System Reform	2021.10.08
34	Notice on Promoting Sanming City's Experience in Hierarchical Diagnosis and Treatment and the Construction of Medical Consortiums	2021.10.29
35	Several Policy Measures on Accelerating the Characteristic Development of Traditional Chinese Medicine	2021.02.09
36	Outline of the 14th Five-Year Plan (2021-2025) for National Economic and Social Development and Vision 2035 of the People's Republic of China	2021.03.11
37	Opinions of the State Council on the Division of Key Tasks in the "Report on the Work of the Government"	2021.03.19
38	Key Tasks for Deepening the Healthcare System Reform in 2022	2022.05.04
39	14th Five-Year Plan for National Health	2022.04.27
40	Hold High the Great Banner of Socialism with Chinese Characteristics and Strive in Unity to Build a Modern Socialist Country in All Respects - Report to the 20th National Congress of the Communist Party of China	2022.10.16
41	Implementation Plan for Strengthening Traditional Chinese Medicine Geriatric Health Services	2022.12.30
42	Notice on Establishing a Dynamic Adjustment Mechanism to Accelerate the Construction of Comprehensive Pilot Zones for Primary Health Care	2022.11.17
43	14th Five-Year Plan for National Health Informatization	2022.11.07
44	National Action Plan to Contain Antimicrobial Resistance (2022-2025)	2022.10.25
45	14th Five-Year Plan for the Development of Health Workforce	2022.08.03
46	Implementation Plan for the Outline for Women and Children's Development in China (2021-2030)	2022.04.02
47	Notice on the Implementation of National Basic Public Health Service Programs in 2022	2022.07.05
48	Key Work Points of the Healthy China Initiative in 2022	2022.03.23
49	14th Five-Year Action Plan for the Capacity Enhancement Project of Primary Traditional Chinese Medicine Services	2022.03.08
50	Guiding Opinions on Promoting the High-Quality Development of Family Doctor Contract Services	2022.03.03
51	Notice on Organizing the Application for Central Finance Supported Demonstration Projects for the Reform and High-Quality Development of Public Hospitals	2022.03.01

52	14th Five-Year National Eye Health Plan (2021-2025)	2022.01.04
53	Notice on the 14th Five-Year Plan for Emergency Medical Rescue for Public Health Incidents	2022.12.31
54	Notice on the 14th Five-Year Plan for Food Safety Standards and Monitoring & Evaluation	2022.08.11
55	14th Five-Year Plan for the Development of Traditional Chinese Medicine	2022
56	14th Five-Year Plan for Health Standardization Work	2022
57	Guiding Opinions of the General Office of the State Council on Promoting the High-Quality Development of Disease Prevention and Control	2023.12.25
58	Guiding Opinions on Comprehensively Promoting the Construction of Tight-Knit County Medical and Health Communities	2023.12.29
59	Opinions on Further Improving the Medical and Health Service System	2023.03.23
60	Opinions on Further Deepening Reform to Promote the Healthy Development of the Rural Medical and Health System	2023.02.23
61	Notice on Implementing the "Quality Service at the Grassroots" Campaign and the Three-Year Action for Community Hospital Construction	2023.12.11
62	Implementation Plan for the Cancer Prevention and Control Action of the Healthy China Initiative (2023-2030)	2023.10.30
63	Key Tasks for Deepening the Healthcare System Reform in the Second Half of 2023	2023.07.21
64	Notice on the Implementation of National Basic Public Health Service Programs in 2023	2023.07.06
65	Key Work Points of the Healthy China Initiative in 2023	2023.03.02
66	Notice on Launching Pilot Projects for the Construction of Tight-Knit Urban Medical Groups	2023
67	Key Tasks for Deepening the Healthcare System Reform in 2024	2024
68	Decision of the Central Committee of the Communist Party of China on Further Comprehensively Deepening Reforms and Advancing Chinese Modernization	2024.07.18
69	Sustainable Development Plan for the Comprehensive Elimination of Leprosy Hazards (2024-2030)	2024.01.23
70	National Action Plan for Health Adaptation to Climate Change (2024-2030)	2024.09.05
71	Notice on Launching the 2024 National Cancer Prevention and Treatment Awareness Week Activities	2024.03.29
72	Implementation Plan for the Chronic Respiratory Disease Prevention and Control Action of the Healthy China Initiative (2024-2030)	2024.07.19
73	Guiding Opinions on Strengthening and Improving National Plague Prevention and Control Work	2024.07.15
74	Implementation Plan for the Diabetes Prevention and Control Action of the Healthy China Initiative (2024-2030)	2024.07.15
75	Notice on Conducting Themed Promotional Activities for "World Hepatitis Day" in 2024	2024.07.01
76	National Disease Prevention and Control Action Plan (2024-2025)	2024.05.21
77	Notice on Promoting the Experience of Liangshan Prefecture, Sichuan Province, in the Prevention and Control of Major Infectious Diseases such as HIV/AIDS	2024.05.13
78	Guiding Opinions on Promoting the Construction of Healthy Villages	2024.08.01

79	Notice on the Implementation of National Basic Public Health Service Programs in 2024	2024.09.09
80	National Tuberculosis Prevention and Control Plan (2024-2030)	2024.11.28
81	Notice on the Establishment and Management of Healthy Weight Management Clinics	2025.03.25
82	Opinions on the Construction and Development of Interdisciplinary Strategic Talents in Medicine, Prevention, and Management	2025.05.12
83	Notice on Issuing the Action Plan for the Prevention and Treatment of Viral Hepatitis in China (2025–2030)	2025.09.03
84	Guiding Opinions on Strengthening Health Management Services for Chronic Diseases at the Primary Care Level	2025.10.24
85	Guiding Opinions on Strengthening the Construction of Specialized Departments in Primary Healthcare Institutions	2025.12.11
